# Level of burnout and associated factors among healthcare workers in central Uganda: A facility-based cross-sectional study

**DOI:** 10.1371/journal.pone.0309701

**Published:** 2024-10-29

**Authors:** Amir Kabunga, Eustes Kigongo, Marvin Musinguzi, Raymond Tumwesigye, Anne Ruth Akello, Walter Acup, Mary Gorretti Asiimwe, Viola Nalwoga

**Affiliations:** 1 Faculty of Medicine, Lira University, Lira, Uganda; 2 Faculty of Public Health, Lira University, Lira, Uganda; 3 Faculty of Medicine, Mbarara University of Science and Technology, Mbarara, Uganda; 4 Faculty of Nursing and Midwifery, Lira University, Lira, Uganda; KSAU-HS: King Saud bin Abdulaziz University for Health Sciences, EGYPT

## Abstract

**Background:**

Burnout among healthcare workers is a global concern with significant implications for both the well-being of the workforce and the quality of patient care. This facility-based cross-sectional study aimed to identify factors associated with burnout among healthcare workers in central Uganda.

**Methods:**

The study, conducted between June and July 2023, utilized a cross-sectional design involving physicians, nurses, and technicians. The study covered both public and private hospitals in central Uganda, incorporating urban and rural settings. A sample size of 550 healthcare workers was selected using a simple random sampling. Data collection involved a socio-demographic survey, the Professional Quality of Life (ProQOL-5). Descriptive statistics, Pearson Chi-square test, and ordinal regression models were employed to analyze demographic factors associated with burnout.

**Results:**

Of 548 participants, 218(39.8%) experienced high burnout levels. Factors significantly associated with high burnout levels included having over 10 years of work experience (OR: 2.04, 95% CI: 1.12–3.73), working more than 40 hours per week (AOR: 4.46, 95% CI: 1.20–16.62), lack of management support (AOR: 14.45, 95% CI: 3.83–54.56), not experiencing workplace violence (AOR: 2.22, 95% CI: 1.31–3.76), and reporting inadequate sleep (AOR: 6.96, 95% CI: 3.86–12.57).

**Conclusion:**

Addressing burnout among healthcare workers in central Uganda requires targeted interventions tailored to the specific challenges faced in the region, including workload distribution, managerial support enhancement, violence prevention strategies, and promotion of adequate sleep. Urgent attention to these factors is essential for enhancing the well-being of healthcare professionals and maintaining quality patient care.

## Introduction

Globally, healthcare systems face challenges related to workforce burnout, affecting the quality of patient care and the overall well-being of professionals [1]. In sub-Saharan Africa, including Uganda, unique contextual factors contribute to the prevalence of burnout among healthcare workers [2]. Limited resources, understaffing, and infrastructure constraints are common challenges faced by healthcare systems in the region [3]. In Uganda, studies highlight the increasing recognition of burnout’s impact on healthcare workers and its potential to compromise the effectiveness of healthcare delivery [4, 5]. Despite the growing awareness, there is a need for more insights into the factors contributing to burnout in specific contexts, such as Central Uganda, to inform targeted interventions and policy changes.

Burnout is a state of extreme fatigue resulting from prolonged exposure to stress [6]. It is distinguished by three specific syndromes: emotional exhaustion, depersonalization, and reduced personal accomplishment [6]. Individuals experiencing burnout often exhibit signs of physical and emotional overextension, a sense of cynicism and callousness towards their work, and a diminished level of professional efficacy [7]. The most recent edition of the International Classification of Diseases-11 has redefined burnout as a workplace phenomenon, emphasizing the involvement of the aforementioned syndromes rather than attributing it to challenges in managing broader life circumstances [8]. The consequences of burnout on healthcare workers are profound and multifaceted [9]. Numerous studies have established a direct correlation between burnout and reduced job satisfaction, increased turnover rates, and compromised patient safety [10]. Burnout negatively impacts healthcare professionals’ mental and physical well-being, leading to higher levels of stress, fatigue, and emotional exhaustion [10]. Additionally, burnout is associated with decreased productivity, impaired professional relationships, and an elevated risk of medical errors [11]. The pervasive effects of burnout extend beyond the individual healthcare worker to impact the overall functioning of healthcare systems, emphasizing the urgency of addressing this issue comprehensively.

Existing literature on burnout among healthcare workers provides valuable insights into the global prevalence and contributing factors [12]. Studies have identified workload, lack of organizational support, and exposure to workplace violence as common factors associated with burnout [13–15]. However, there is a notable gap in the literature regarding the specific dynamics of burnout in the context of Central Uganda. Localized studies are essential to understanding how regional challenges, including resource limitations, cultural factors, and unique healthcare system constraints, contribute to burnout. Furthermore, there is a need for research that assess the relationship between demographic factors, work-related variables, and psychosocial aspects in Central Uganda’s healthcare workforce to inform targeted interventions.

The Job Demands-Resources (JD-R) model is a framework that provides insights into the relationship between job characteristics and employee well-being, particularly in the context of burnout among healthcare workers. This model posits that every work environment has its unique combination of job demands and job resources [16]. Job demands are aspects of the job that require physical, psychological, or emotional effort and are associated with physiological and psychological costs [16]. Examples in healthcare settings might include high patient loads, time pressure, and emotionally demanding interactions with patients and their families [17]. On the other hand, job resources are the physical, psychological, social, or organizational aspects of the job that help achieve work goals, reduce job demands, and stimulate personal growth and development [16]. These resources might include social support from colleagues, opportunities for training and development, and autonomy in decision-making. In this study, the JD-R model can provide a lens through which to understand how specific job demands and resources in this setting might contribute to burnout [17].Healthcare workers in Uganda are facing high job demands such as understaffing, long working hours, violence, sleeplessness, and limited access to necessary resources, which could lead to increased burnout levels [4]. Conversely, if they have adequate job resources such as supportive colleagues, training opportunities to enhance skills, a sense of autonomy in their roles, and resilience gained through experience, it could mitigate the risk of burnout [18]. Integrating factors such as violence, sleeplessness, and experience into the JD-R model discussion enhances the understanding of the relationship between job demands and resources contributing to burnout among healthcare workers in central Uganda.

Recent studies indicate a concerning rise in mental health rates among healthcare workers in Uganda [4, 19]. This sheds light on new challenges faced by the workforce. Uganda’s health system is confronted by numerous challenges. It remains underfunded, significantly below the recommended 15% budget allocation according to the Abuja Declaration, of which Uganda is a signatory [20]. Between 2010 and 2016, the health sector’s budget averaged 7.8% of the national budget [21]. However, in the 2020/2021 financial year, it dropped to 5.1% from the previous year’s 7.9% [20] (Ministry of Finance, Planning and Economic Development, 2020). Other significant hurdles include health workers being severely underpaid, shortages of personnel, inadequate supplies of medicines and essential equipment in government facilities, limited hospital beds, high costs, and poor accessibility to health services [22]. All of which exacerbate the risk of burnout among the healthcare workforce [19]. This situation has led to a burgeoning crisis of burnout, jeopardizing the well-being of individual professionals and undermining the efficiency and quality of healthcare delivery.

The contextual factors contributing to burnout among healthcare workers in central Uganda, such as resource limitations, high patient-to-staff ratios, and cultural expectations, are significant. Studies have shown that these factors exacerbate stress and burnout [18]. Addressing these unique local challenges is crucial for effective intervention and support. Studies conducted by Udho & Kabunga (2022) [4] and Amir & Okalo (2022) [11], have provided valuable insights into the overall prevalence and contributing factors of burnout. However, these studies were limited in scope, primarily focusing on nurses and neglecting other healthcare professionals. Therefore, our study aims to expand upon this existing literature by identifying specific factors associated with burnout among all healthcare workers in central Uganda. Through our research, we aim to provide actionable recommendations such as continuous training programs, clear guidelines on workload management, peer support groups, and culturally sensitive counseling services within healthcare facilities to address these challenges comprehensively.

## Materials and methods

### Study design and settings

Conducted between June and July 2023, this cross-sectional analytical study took place in five health facilities in central Uganda. The design was chosen for this study to provide a snapshot of the prevalence of burnout and associated factors among healthcare workers in central Uganda. This design allows for the simultaneous collection of data from a diverse group of healthcare workers at a specific point in time. The investigation covered diverse healthcare environments throughout central Uganda. Central Uganda, located in the heart of East Africa, incorporates urban and rural landscapes, including the capital city, Kampala. The was selected due to several factors. Firstly, central Uganda represents a geographically diverse region with a mix of urban and rural healthcare facilities. This diversity allows for a broader representation of healthcare workers and enhances the generalizability of the study findings to similar settings in Uganda. Additionally, central Uganda is home to a significant proportion of the country’s population and healthcare facilities, making it an ideal location to assess the prevalence of burnout among healthcare workers and identify potential factors contributing to this phenomenon.

### Study participants

The participants, consisting of physicians, nurses, and technicians actively engaged in direct patient care and holding key roles within the central Uganda health system, were selected from diverse medical facilities in the region. The inclusion criteria required participants to be actively practicing physicians, nurses, or technicians involved in direct patient care within the central Uganda health system. Exclusion criteria was individuals on extended leave and those with less than one year of experience. These criteria were established to ensure a comprehensive understanding of the potential impact of burnout on quality of life.

### Sample size determination

To determine the sample size, a 95% confidence interval (CI) and a 5% margin of error were used for this cross-sectional study. The estimated proportion (P) was set at 50%, and the sample size was increased to 550 healthcare workers, considering a 30% non-response rate. Simple random sampling was employed, with participants selected from five prominent hospitals in central Uganda. Each hospital received an equal allocation of the total sample, and proportionate sampling was applied for each job category within every hospital. This approach aimed to ensure a representative sample that reflects the diverse roles and experiences of healthcare workers in central Uganda.

### Data collection tools and methods

Data was collected through face-to-face interviews by trained research assistants. Data collection involved two tools: the socio-demographic survey and the English version of the Professional Quality of Life (ProQOL-5). The socio-demographic survey had items like age, gender, marital status, work experience, Weekly work load, job category, Weekly work load, management support, and experience workplace violence. The ProQOL is a self-report questionnaire with 30 items designed to evaluate burnout (10 items), compassion fatigue (10 items), and compassion satisfaction (10 items). Burnout was measured using subscale of the ProQOL, which consists of 10 items. The ProQOL-5, version of the scale encompasses three subscales: burnout, compassion fatigue, and compassion satisfaction, each consisting of 10 items [23]. In this study, we focused on measuring burnout, utilizing the burnout subscale, which comprises 10 items. A Burnout score of 22 and below indicates a low level, 23–41 suggests an average level, and 42 or higher signifies a high level of burnout [23]. The ProQOL has been validated for periodic self-monitoring [24]. Moreover, studies have utilized the ProQOL to diagnose and classify burnout among healthcare [25]. While the ProQOL has been previously utilized in various studies, including ones involving healthcare workers [4, 5, 26], its specific validation for the Ugandan context has not been extensively documented. To ensure the appropriateness of the ProQOL in identifying and grading burnout among healthcare workers in Uganda, we conducted a pilot study with a sample of healthcare professionals from multiple facilities in the central region. Based on the results of this pilot study, we found that the ProQOL demonstrated good reliability recorded as 0.89 for assessing burnout among healthcare workers in our setting.

### Study procedure

After obtaining ethical approval, five research assistants, each with research experience, underwent training on data collection tools, study objectives, and ethical considerations. Collaboration with officers from various health facilities in central Uganda, including public and private, rural and urban establishments, was established. Participants were briefed on the study’s objectives and were encouraged to fill out a self-administered questionnaire, which took approximately 26 minutes to complete on average. Data collection involved two tools: the socio-demographic survey and the ProQOL-5. The socio-demographic survey included items like age, gender, marital status, work experience, weekly workload, job category, management support, and experience of workplace violence. We ensured bias control by training research assistants extensively on unbiased data collection techniques and using standardized tools. A trained psychologist was available for assistance to mitigate any distress during the survey process. The use of the ProQOL-5 tool, specifically its burnout dimension, allowed for a comprehensive assessment while focusing on our primary research objective.

### Ethical approval

Adhering to ethical standards, our study protocol received approval from the Lira University Ethics Committee (LUREC 2023–24). This demonstrates our dedication to safeguarding the participants’ wellbeing, rights, and privacy. During this procedure, written informed consent was secured, ensuring participants had complete awareness of the study’s objectives, possible risks, and benefits. Confidentiality was rigorously maintained throughout the study. Each participant was assigned a unique identifier, and all data were stored securely in a password-protected electronic database accessible only to the research team. Personal identifying information, such as names and contact details, was kept separate from the main dataset. Only authorized personnel had access to the raw data, and any published results were presented in aggregate form to prevent individual identification. Only aggregated data were used for analysis, and individual responses were not identifiable in any publication or presentation. Results were reported in a way that prevented the identification of specific participants or facilities, further safeguarding confidentiality.

### Data management and analysis

The data were analyzed using STATA software version 17 through univariable, bivariable, and multivariable analyses. Initially, descriptive statistics were conducted for both the dependent variable (burnout) and independent variables (demographic factors). Simple frequencies and percentages were presented in a frequency distribution table. At the bivariable level, a Pearson Chi-square test was employed to ascertain whether associations existed between burnout and demographic factors. Subsequently, variables that exhibited significance at the 25% level [27] in the bivariable analysis were included in the multivariable analysis, which encompassed the use of an ordinal regression model. The likelihood ratio test and Brant test were executed to evaluate the proportionality assumption and determine whether to apply the proportional ordered model (POM) or partial proportional ordered model (PPOM). The stepwise ordinal logistic procedure was implemented, and the results were presented as odds ratios with corresponding 95% confidence intervals [28]. Finally, the goodness of fit was assessed through the Hosmer test to evaluate the chosen predictive model. To address potential biases, measures such as random sampling from multiple healthcare facilities and adjusting for confounding variables in regression models were undertaken.

## Results

### Demographic factors

A total of 548 participants fully participated in the study. Table 1 indicates that approximately half of the participants, specifically 279 (50.9%), were below the age of 29. A majority of the participants, accounting for 340(62.0%), were female, while 352 (64.2%) reported living with a spouse or partner, and 242(44.2%) had worked for less than five years. In terms of occupation, the majority of participants were nurses, comprising 255(46.5%), followed by technicians at 190 (34.7%). More than half of the participants, totaling 318 individuals (58.0%), reported receiving support from management, and a majority of 343(62.6%) did not experience workplace violence. Additionally, most participants, 312(56.9%), reported having adequate sleep at night.

**Table 1:** Demographic characteristics of healthcare workers in central Uganda (n = 548).

Variable	Frequency (n)	Percentage (%)
**Age**		
<29 years	279	50.9
29–38 years	193	35.2
>39 years	76	13.9
**Gender**		
Female	340	62.0
Male	208	38.0
**Marital status**		
With spouse/partner	352	64.2
Without spouse/partner	196	35.8
**Work experience**		
<5 years	242	44.2
5–10 years	140	25.6
<10 years	166	30.3
**Job category**		
Physician	190	34.7
Nurse	255	46.5
Technician	103	18.8
**Weekly work load**		
30–38 hours	283	51.6
40 hours and above	265	48.4
**Receive management support**		
Yes	318	58.0
No	230	42.0
**Experience workplace violence**		
Yes	205	37.4
No	343	62.6
**Have adequate sleep at night**		
Yes	312	56.9
No	236	43.1

### Levels of burnout

Burnout was assessed using the professional quality of life tool, following the guidelines provided by Hegarty [29]. The tool classifies burnout into three categories based on scores: low (less than 22), average (22–41), and high level (more than 41). According to Table 2, the majority of participants, 218(39.8%), exhibited a high level of burnout, while only 181(33.0%) had a low level of burnout.

**Table 2:** Levels of burnout for healthcare workers in central Uganda (n = 548).

Burnout	Frequency (%)	95% Confidence Interval
Low	181(33.0)	29.2–37.1
Average	149(27.2)	23.6–31.1
High	218(39.8)	35.8–44.0
**Total**	**548(100)**	

### Factors associated with burnout among healthcare workers

Table 3 presents the results of a multivariate proportional ordered model (POM) that involved all nine variables found to be significant at the bivariate level (p < 0.05), utilizing the backward elimination technique. Among these variables, including work experience, job category, weekly work load, having management support, experiencing workplace violence, and sleep quality, there were associations observed with burnout levels, as evidenced by a p-value less than 0.05. However, upon further analysis, it was found that job category, management support, and workplace violence exhibited a significant Brant test score (p < 0.05). This suggests a violation of the proportional assumption as described by McCullagh [30]. Consequently, a partial proportional ordered model was implemented to account for this violation.

**Table 3:** Proportional ordered model (POM) using sociodemographic factors.

Variable	Levels of Burnout				POM	
	Low n(%)	Average n(%)	High n(%)	^x^P value	OR(95% CI)	Brant test
**Age**						
<29 years	89(49.2)	88(59.1)	102(46.8)			
29–38 years	21(11.6)	60(40.3)	112(51.4)	<0.001		
>39 years	71(39.2)	1(0.7)	4(1.8)			
**Gender**						
Female	130(71.8)	81(54.4)	129(59.2)	0.003		
Male	51(28.2)	68(45.6)	89(40.8)			
**Have a spouse**						
Yes	135(74.6)	82(55.0)	135(61.9)	0.001		
No	46(25.4)	67(45.0)	83(38.1)			
**Work experience**						
<5 years	87(48.1)	71(47.6)	84(38.5)		Ref	
5–10 years	20(11.1)	45(30.2)	75(34.4)	<0.001	0.77(0.41, 1.45)	0.417
<10 years	74(40.9)	33(22.2)	59(27.1)		1.73(0.98, 3.04)	0.057
**Job category**						
Technician	98(54.1)	1(0.7)	4(1.8)		Ref	
Nurse	35(19.3)	77(51.7)	143(65.6)		0.02(0.01, 0.05)	<0.001
Physician	48(26.5)	71(47.7)	71(32.6)		0.02(0.01, 0.06)	<0.001
**Weekly work load**						
30–38 hours	167(92.3)	30(20.1)	86(39.5)	<0.001	Ref	
40 hours and above	14(7.7)	119(79.9)	132(60.6)		4.00(1.75, 9.14)	0.001
**Management support**						
Yes	175(96.7)	29(19.5)	114(52.3)	<0.001	Ref	
No	6(3.3)	120(80.5)	104(47.7)		3.97(1.77, 8.94)	<0.001
**Workplace violence**						
Yes	3(1.7)	90(60.4)	112(51.4)	<0.001	Ref	
No	178(98.3)	59(39.6)	106(48.6)		2.46(1.65, 3.69)	<0.001
**Adequate night sleep**						
Yes	31(17.1)	98(65.8)	183(83.9)	<0.001	Ref	
No	150(82.9)	51(34.2)	35(16.1)		5.36(3.41, 8.43)	<0.001

OR: odds ratio, CI: confidence interval,

*p<0.05,

^x^P value of Chi-square test

### Partial proportional ordered regression model using sociodemographic factors

Table 4 displays the results of a partial proportional ordered regression model conducted using the "gologit2" STATA embedded command and the "autofit" option. This approach relaxed the parallel line constraint for variables that did not adhere to the proportionality assumption, as outlined by Williams [28]. The Hosmer goodness of fit test reported a non-significant p-value (0.221), suggesting a suitable model selection. The model specifically compared individuals with a high burnout level against those with low and average levels combined. According to the findings, healthcare workers with more than 10 years of service were twice as likely to experience high burnout compared to those with less than five years of experience (OR: 2.04, 95% CI: 1.12–3.73, p = 0.020). Additionally, individuals working more than 40 hours per week were 4.46 times more likely to suffer from high burnout compared to those working fewer hours (AOR: 4.46, 95% CI: 1.20–16.62, p = 0.026). Furthermore, healthcare workers who reported not receiving management support were significantly more at risk, being 14.45 times more likely to suffer from high burnout compared to those who had received management support (AOR: 14.45, 95% CI: 3.83–54.56, p<0.001). Those who did not experience workplace violence were 2.22 times more likely to exhibit high burnout levels compared to those who reported having experienced workplace violence (AOR: 2.22, 95% CI: 1.31–3.76, p = 0.003). Lastly, individuals reporting inadequate sleep time were 6.96 times more likely to experience high burnout compared to those reporting adequate sleep (AOR: 6.96, 95% CI: 3.86–12.57, p<0.001).

**Table 4:** Partial proportional ordered regression model using sociodemographic factors.

Variables	Odds Ratio	95% Confidence Interval	P value
**Work experience**			
<5 years	Reference		
5–10 years	1.12	0.56–2.23	0.754
>10 years	2.04	1.12–3.73	0.020
**Job category**			
Technician	Reference		
Nurse	0.47	0.12–1.81	0.271
Physician	0.56	0.14–2.22	0.411
**Weekly work load**			
30–38 hours	Reference		
40 hours and above	4.46	1.20–16.62	0.026
**Management support**			
Yes	Ref		
No	14.45	3.83–54.56	<0.001
**Experience workplace violence**			
Yes	Reference		
No	2.22	1.31–3.76	0.003
**Adequate night sleep**			
Yes	Reference		
No	6.96	3.86–12.57	<0.001

## Discussion

The study investigated factors associated with burnout among healthcare workers in central Uganda. Results indicated that 39.8% of participants experienced high burnout levels. Factors significantly associated with high burnout levels included having over 10 years of work experience (OR: 2.04, 95% CI: 1.12–3.73), working more than 40 hours per week (AOR: 4.46, 95% CI: 1.20–16.62), lack of management support (AOR: 14.45, 95% CI: 3.83–54.56), not experiencing workplace violence (AOR: 2.22, 95% CI: 1.31–3.76), and reporting inadequate sleep (AOR: 6.96, 95% CI: 3.86–12.57).

Our research findings on burnout levels among healthcare workers in central Uganda, revealing that 39.8% are experiencing a high level of burnout, emphasize the critical issue of occupational stress within the healthcare sector. This aligns with a growing body of literature consistently identifying healthcare worker burnout as a widespread problem [4, 31]. Numerous studies have pointed out the association between high workload, inadequate resources, and increased burnout among healthcare professionals [1]. However, it is essential to consider the unique contextual factors in central Uganda that may contribute to burnout, such as specific challenges faced by the healthcare system in that region. Cultural expectations regarding work ethic, community reliance on healthcare providers, and the stigma surrounding mental health issues may also play significant roles [18]. Disparities in resource allocation, insufficient staffing, and limited access to mental health support could exacerbate burnout [13]. The results indicate an urgent need for targeted interventions and policy changes to address the root causes of burnout among healthcare workers in central Uganda, ultimately enhancing both the well-being of the workforce and the quality of patient care.

Our findings show that healthcare workers with more than 10 years of service were twice as likely to experience high burnout compared to those with less than five years of experience. This result suggests that the cumulative stress and challenges associated with longer service tenure may contribute to an increased risk of burnout among healthcare professionals. One key aspect to consider in this finding is the potential impact of increased job demands and responsibilities that come with seniority. Healthcare workers with more years of experience may find themselves in leadership roles, dealing with administrative duties, and facing higher expectations, all of which can contribute to heightened stress levels. The cumulative nature of these stressors over time may explain the higher prevalence of burnout among individuals with more extended periods of service. Additionally, these individuals may have witnessed significant changes in the healthcare system over the years, such as resource constraints and shifting patient demographics, factors that can contribute to burnout. This finding aligns with a trend observed in various studies on healthcare worker burnout [32]. While experience may bring expertise, it can also expose healthcare workers to additional stressors that contribute to burnout. Therefore, organizations need to implement targeted interventions, such as regular mental health assessments, counseling services, and training programs, to address the specific needs of healthcare workers with longer service tenure.

Our results show that individuals working more than 40 hours per week in central Uganda are 4.46 times more likely to experience high burnout aligns with existing literature on healthcare worker burnout [32]. Numerous studies have consistently demonstrated a positive association between longer working hours and increased burnout among healthcare professionals [33]. This result is in harmony with research that highlights the detrimental effects of extended working hours on both physical and mental well-being [10]. The increased risk of burnout observed in individuals working over 40 hours per week shows the importance of addressing workload issues and implementing strategies such as workload distribution, proper scheduling, and promoting a healthy work-life balance in the healthcare sector. It also emphasizes the need for interventions and policies aimed at mitigating the impact of excessive working hours on healthcare workers’ mental health and overall job satisfaction, ultimately contributing to the well-being of the healthcare workforce in central Uganda.

Our research reveals a significant risk for healthcare workers who do not receive support from management, as they are 14.45 times more likely to experience high burnout compared to those who receive such support. This result reveals a crucial connection between the absence of management support and an elevated risk of burnout, aligning with existing literature emphasizing the pivotal role of organizational support in mitigating burnout among healthcare workers globally. Numerous studies across different contexts consistently show that a supportive management environment, encompassing resources, communication, and emotional assistance, acts as a protective factor against burnout [34]. Our findings highlight the considerable vulnerability of healthcare workers lacking adequate management support, emphasizing a pressing concern that requires attention. Conversely, our results align with research suggesting that interventions promoting management support can effectively reduce burnout rates among healthcare professionals [34]. The outcomes of our study underscore the urgent need for healthcare organizations in central Uganda to prioritize and enhance their managerial support structures. Addressing this issue is crucial for maintaining a resilient and motivated healthcare workforce in the region.

Our finding shows that healthcare workers in central Uganda who did not experience workplace violence were 2.22 times more likely to exhibit high burnout levels is fascinating and somewhat counterintuitive, given the prevailing literature on healthcare worker burnout. The unexpected finding that healthcare workers in central Uganda who did not experience workplace violence were more likely to exhibit high burnout levels could be due to several factors. One potential explanation is that in environments with no reported workplace violence, there might be underreporting or normalization of stressful events. Numerous studies in various contexts have consistently associated workplace violence with increased burnout among healthcare professionals [35]. Further investigation into the cultural and organizational dynamics surrounding workplace violence reporting and support systems in central Uganda could shed more light on this paradoxical relationship. While existing literature highlights the detrimental impact of violence on healthcare workers’ well-being [36], this study suggests the need for considerations tailored to the local context.

Lastly, our findings reveal a significant association between inadequate sleep time and a higher likelihood of experiencing burnout. This aligns with existing literature, as numerous studies have consistently identified the crucial role of sleep in mitigating burnout among healthcare professionals [37]. Research from various regions has highlighted the impact of insufficient sleep on mental and physical well-being, with sleep deprivation being linked to increased stress, decreased job satisfaction, and ultimately higher burnout rates [38]. The results signify the substantial risk, emphasizing the urgency of addressing sleep-related issues to prevent burnout in this specific population. These findings indicate the importance of implementing targeted interventions, such as workplace policies promoting adequate rest and fostering a supportive environment for healthcare workers. Addressing sleep-related factors can potentially contribute to a reduction in burnout rates, improving overall job satisfaction and, consequently, the quality of patient care.

### Strengths and limitations

The study adopts a comprehensive approach by including healthcare professionals from various categories working in both public and private hospitals in central Uganda. This diverse representation enhances the generalizability of the findings to the broader healthcare workforce in the region. The use of the Professional Quality of Life (ProQOL-5) tool, a well-established instrument for assessing burnout, enhances the validity and comparability of the study’s results.

However, the study has its limitations. The cross-sectional design employed restricts the ability to establish causal relationships between identified factors and burnout. To gain a more nuanced understanding of the temporal dynamics of these associations, longitudinal studies would be necessary. Additionally, the reliance on self-reported data, including responses to the ProQOL-5 tool, introduces the potential for response bias and social desirability bias. While the study offers valuable insights into burnout in central Uganda, caution is warranted when generalizing the findings to other regions with different contextual factors.

### Practical implications

Based on the study findings, there is a need for increased hiring in healthcare facilities in central Uganda to distribute workloads more evenly. This would help reduce stress and exhaustion among staff. Additionally, there is a need to implement mental health support services. These services could include regular counseling sessions, access to mental health professionals, and creating a supportive environment where staff feel comfortable seeking help. The health sector could provide training workshops on stress management, resilience building, and coping strategies for healthcare workers. Equipping them with these tools could better prepare them to handle the pressures of their jobs. Offering more flexible scheduling options for healthcare workers, such as part-time positions, job sharing, or flexible hours, can help staff better balance work and personal life, ultimately reducing burnout.

## Conclusion

Overall, the study emphasizes the critical need for healthcare organizations in central Uganda to urgently implement targeted strategies. These strategies include continuous training programs, the establishment of clear guidelines on workload management and distribution, the formation of peer support groups, and the implementation of culturally sensitive counseling services within healthcare facilities. These measures are essential for addressing the identified factors contributing to burnout among healthcare workers, thereby promoting the well-being of the workforce and enhancing the quality of patient care. For future studies, refining the research questions to explore how specific organizational factors, such as workload distribution and managerial support, contribute to varying levels of burnout among healthcare workers in central Uganda could provide deeper insights. Future research also, might consider employing longitudinal designs to track changes in burnout levels over time, allowing for a more dynamic understanding of the factors influencing burnout among healthcare workers in central Uganda.

## Supporting information

S1 Data (DTA)
